# Winter wheat ear counting based on improved YOLOv7x and Kalman filter tracking algorithm with video streaming

**DOI:** 10.3389/fpls.2024.1346182

**Published:** 2024-06-17

**Authors:** Xingmei Xu, Lei Zhou, Helong Yu, Guangyao Sun, Shuaipeng Fei, Jinyu Zhu, Yuntao Ma

**Affiliations:** ^1^ College of Information and Technology, Jilin Agricultural University, Changchun, Jilin, China; ^2^ State Key Laboratory of Vegetable Biobreeding, Institute of Vegetables and Flowers, Chinese Academy of Agricultural Sciences, Beijing, China; ^3^ College of Land Science and Technology, China Agricultural University, Beijing, China

**Keywords:** wheat ear counting, real-time detection, YOLOv7x, Kalman filter, UAV

## Abstract

Accurate and real-time field wheat ear counting is of great significance for wheat yield prediction, genetic breeding and optimized planting management. In order to realize wheat ear detection and counting under the large-resolution Unmanned Aerial Vehicle (UAV) video, Space to depth (SPD) module was added to the deep learning model YOLOv7x. The Normalized Gaussian Wasserstein Distance (NWD) Loss function is designed to create a new detection model YOLOv7xSPD. The precision, recall, F1 score and AP of the model on the test set are 95.85%, 94.71%, 95.28%, and 94.99%, respectively. The AP value is 1.67% higher than that of YOLOv7x, and 10.41%, 39.32%, 2.96%, and 0.22% higher than that of Faster RCNN, SSD, YOLOv5s, and YOLOv7. YOLOv7xSPD is combined with the Kalman filter tracking and the Hungarian matching algorithm to establish a wheat ear counting model with the video flow, called YOLOv7xSPD Counter, which can realize real-time counting of wheat ears in the field. In the video with a resolution of 3840×2160, the detection frame rate of YOLOv7xSPD Counter is about 5.5FPS. The counting results are highly correlated with the ground truth number (R^2 = ^0.99), and can provide model basis for wheat yield prediction, genetic breeding and optimized planting management.

## 1 Introduction

Wheat is one of the most important food crops in the world with a global production of 785 million tons in 2023 (FAO, 2023). Wheat production has a direct impact on world food security. The number of wheat ears per unit area is one of the main determinants of wheat yield (Li L. et al., 2022). Wheat ear counting is a labor-intensive work. Timely, accurate, and high-throughput acquisition of wheat ear information is crucial to improve wheat productivity (Jin et al., 2022; Zhao et al., 2022; Zhu et al., 2022).

Computer vision and machine learning algorithms that combine color, texture and morphological features of wheat ears have been able to count wheat ears (Li et al., 2017; Fernandez-Gallego et al., 2018; Tan et al., 2020), but the generalization performance of this method is weak. When used in different scenarios, the algorithm needs to be changed accordingly, which does not meet the real-time detection of wheat ears in the field with complex environment. With the improvement of computer performance, image recognition algorithms have been widely used based on deep learning. Detection and counting of wheat ears can be achieved by image segmentation (Misra et al., 2020; Sanaeifar et al., 2023) and object detection (Hasan et al., 2019; Sadeghi-Tehran et al., 2019; Xiong et al., 2019), thus wheat yield can be estimate accordingly.

Object detection algorithms can be divided into two categories: single-stage and two-stage. Single-stage object detection algorithms, such as Single Shot Detector (SSD) (Liu et al., 2016) and You Only Look Once (YOLO) series (Li C. et al., 2022; Wang et al., 2022a), can directly obtain the location and category information of the object from the image, and do not need to generate region proposal information. Two-stage object detection algorithms usually consist of generating and classifying candidate boxes. Representative algorithms include Fast RCNN (Girshick, 2015) and Faster RCNN (Ren et al., 2017). Liu et al (Liu et al., 2022) proposed a dynamic color transformation network to reduce false negatives and to improve the wheat ear detection by modifying the color channel of the input image. Zhao et al (Zhao et al., 2022) added angle information to the detection results, and introduced the orientation information of wheat ears into the YOLOv5 model to effectively enhance the detection performance of wheat ears under occlusion conditions. A micro-scale object detection layer is added to the YOLOv5 model to improve the wheat ear detection ability based on UAV images. Zang et al (Zang et al., 2022) introduced a channel and a global attention module into YOLOv5s to extract target features more effectively, to suppress useless information, and to achieve better detection results. Faster RCNN is the commonly used detection algorithm in wheat ear counting with two-stage object detection algorithm (Madec et al., 2019; Li L. et al., 2022). However, the performance of Faster RCNN is weak in the detection of small objects (Eggert et al., 2017). The image segmentation algorithm based on deep learning can accurately find the position and edge of the target to realize the recognition and counting of wheat ears (Ma et al., 2020).

Multi-object tracking algorithms based on object detection include Kalman filter (Kalman, 1960), kernelized correlation filter (KCF) (Henriques et al., 2015), multiple hypothesis tracking (MHT) (Reid et al., 1978; Kim et al., 2015), etc. Kalman filter is a linear filter to realize the state transition prediction problem, which can predict the trajectory of moving objects in image sequences. The Sort (Bewley et al., 2016) and DeepSort (Wojke et al., 2017) algorithms are designed by combining Kalman filter and Hungarian matching algorithm (Kuhn, 1955), which can track each object in the video stream in real time. Yang et al (Yang et al., 2022) used CenterNet to establish a target detection model and DeepSort to track targets to realize automatic counting of cotton seedlings, and the counting result R^2^ reached 0.967. In order to quickly estimate tea production, Li et al (Li et al., 2023) modified the YOLOv5 model to improve the detection accuracy of tea buds, and combined the Kalman filter algorithm with the Hungarian matching algorithm to achieve accurate and reliable counting of tea buds. Zhou et al (Zhou et al., 2023) used YOLOv5, ResNet50 and DeepSort models to locate and track the growth and development of individual rice panicles, to determine the heading date, and to analyze the fine phenotypic changes of rice panicle flowering time under different nitrogen fertilizer treatments. For multi-target tracking and counting, the Kalman filter tracking algorithm is more accurate and efficient and is suitable for real-time tracking and counting of multiple targets (Villacrés et al., 2023). Therefore, the object detection algorithm combined with the Kalman filter is an accurate, efficient and reliable method for counting in the video stream.

The UAV image has high resolution with a large number of wheat ears in each frame. The wheat ears account for a small number of pixels in the image. Wheat ear images are obtained from different angles with UAV video stream. Fast tracking of the detected wheat ears is the key to wheat counting under the video stream for real time detection. Therefore, the aims of the current study are: (1) YOLOv7xSPD is constructed based on YOLOv7x model to improve the accuracy of wheat ear detection. (2) YOLOv7xSPD is combined with Kalman filter tracking algorithm and Hungarian matching algorithm to establish a real-time wheat counting under video flow, called YOLOv7xSPD Counter. (3) The wheat detection accuracies are evaluated for six different target detection algorithms.

## 2 Materials and methods

### 2.1 UAV-based wheat image collection

**Figure 1:**
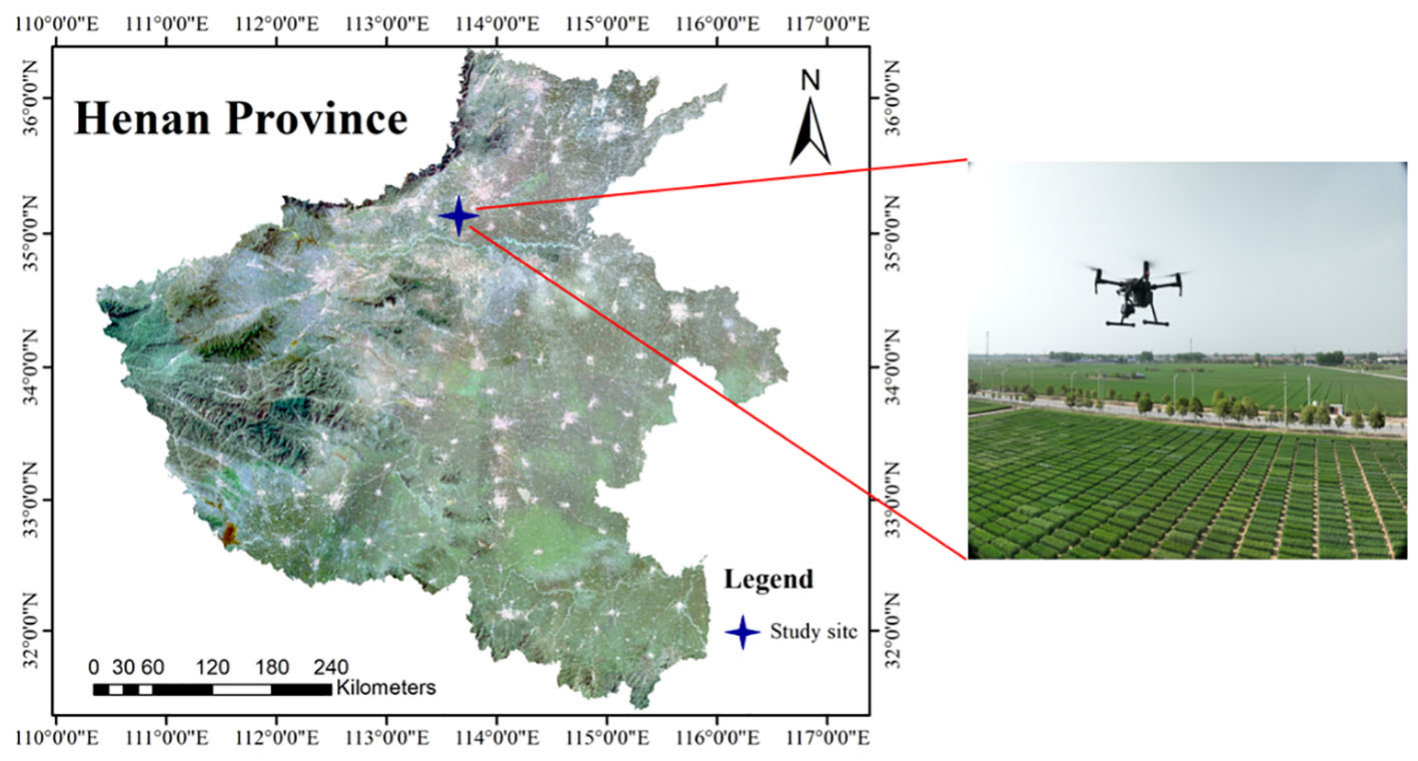
Geographical location of wheat planting area and UAV used for video capture.

The experiment was conducted at a research site of Chinese Academy of Agricultural Sciences (113° 45′ 40′′ E, 35° 8′ 11′′ N) in Xinxiang, Henan province, China (**Figure 1**). The images were collected on April 28, 2023, when winter wheat was at the beginning of grain filling stage. A DJI Mavic3T (DJI, Shenzhen, China) and an integrated 20-megapixel camera was used to capture the video stream with a resolution of 3840×2160 and a frame rate of 30FPS. In order to obtain the detailed video of the wheat canopy, the camera uses 7x zoom and maintains a 90° angle of view to perform the flight mission from 11:00 to 13:00 on a clear day. The flight route is at a constant speed of 0.5 m/s 4 m above the canopy.

### 2.2 Dataset creation

**Figure 2:**
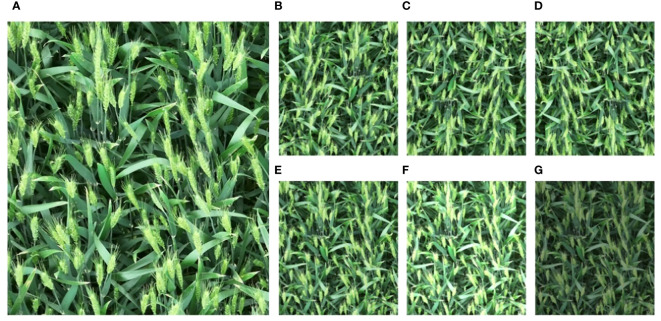
Comparison of original and enhanced images, **(A)** original image, **(B)** horizontal flip, **(C)** vertical flip, **(D)** horizontal and vertical flip, **(E)** gaussian blur, **(F)** increase contrast, **(G)** decrease contrast.

In order to reduce the overlap rate of each image, one frame is extracted every 45 frames. The obtained frame is used as the training image, and the resolution of each image is 3840×2160. To facilitate training, each image was cropped from the middle, resulting in 233 images with a resolution of 1920×2160. LabelMe was used to annotate the original images. In field experiments, objects are subject to variations induced by factors such as lighting conditions, weather changes, and wind speed. In order to improve the generalization performance of the model, six schemes including horizontal flip, vertical flip, horizontal-vertical flip, Gaussian blur, increase and decrease contrast are used to perform data enhancement on the image. Among them, image flipping can help the model train objects from different angles and directions, while Gaussian blur and adjusting contrast can simulate the effects of weather changes. 1631 image data are then obtained. The original image and enhanced effect of the image are shown in **Figure 2**.

**Figure 3:**
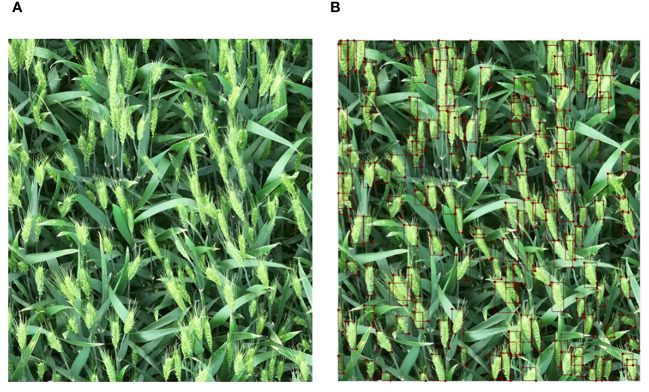
Comparison between the original image and the annotated image, **(A)** the original image and **(B)** the annotated image.

The script written in Python language was used to annotate and convert the enhanced image to obtain its corresponding annotation file. **Figure 3** shows a schematic diagram of the annotated file. 1470 images were selected for model training and 161 images were used for model testing. The data used for model training was divided into training set and validation set according to the ratio of 9:1. In the image dataset, the average number of objects in each image is about 139.1, and the total number of objects is 226,916. A total of 20 videos are used for testing, with a resolution of 3840×2160. Since the videos captured by the UAV will pass through the open land without wheat cultivation, the number of wheat ears in each video is distributed between 300 and 700. The number of wheat ears in the video was counted by three persons, and the counting error for each video was between 1 and 3. The average number of these three persons was taken as the ground true number of wheat ears.

### 2.3 Construction of the wheat ear detection model

#### 2.3.1 YOLOv7xSPD

**Figure 4:**
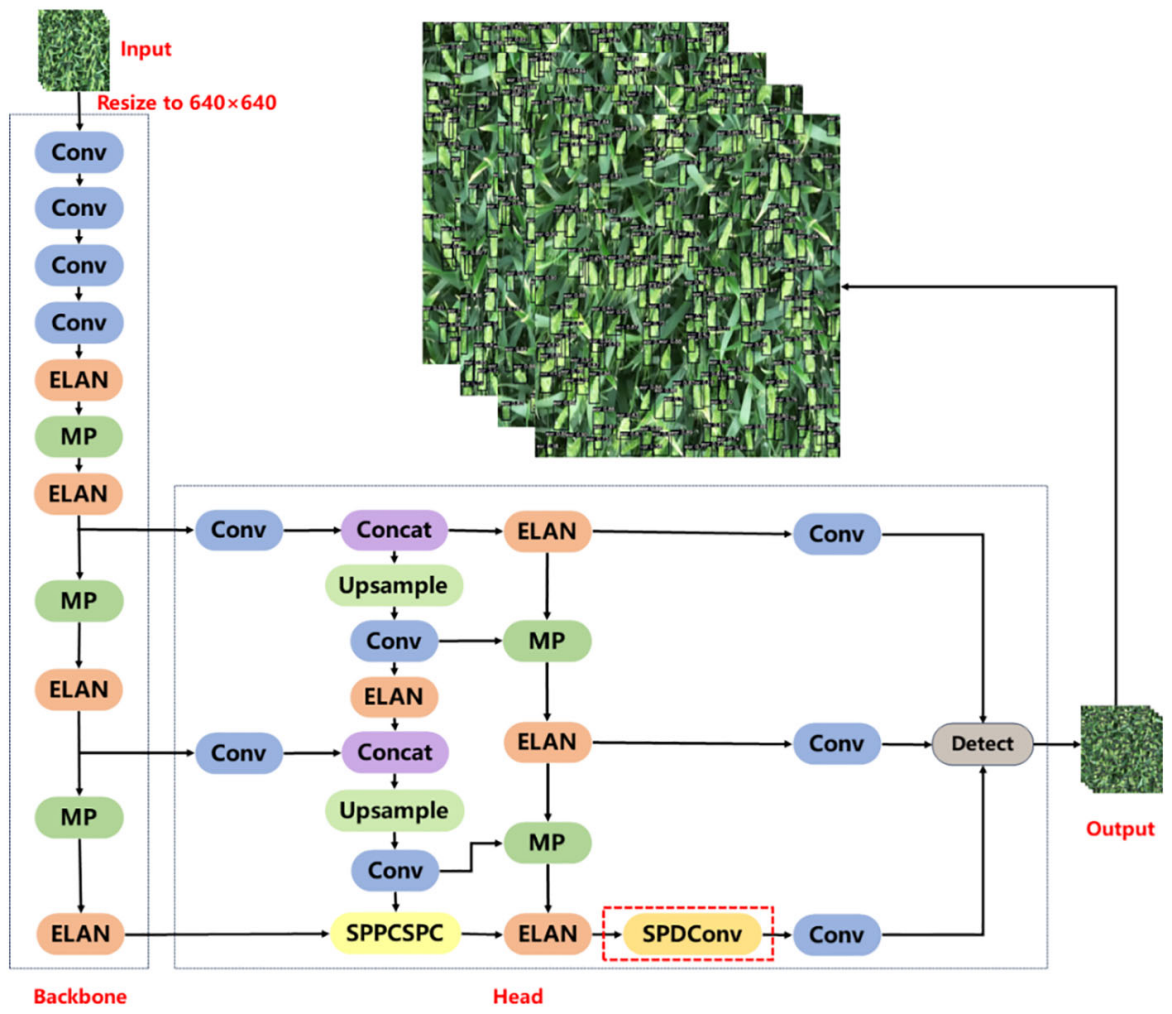
Structure diagram of YOLOv7xSPD network. The red dashed box is the SPDConv module.

YOLOv7xSPD was constructed using YOLOv7x as the basic model. YOLOv7x is obtained from YOLOv7 by scaling the depth and width of the entire model. YOLOv7x consists of two parts: Backbone and Head. The size of wheat ears under large-resolution images is very small, and adding Space to depth Conv (SPDConv) (Sajjadi et al., 2018) module at the end of the Head part of YOLOv7x can enhance the accuracy of the model for small-size target detection. **Figure 4** shows the network structure diagram of YOLOv7xSPD model.

**Figure 5:**
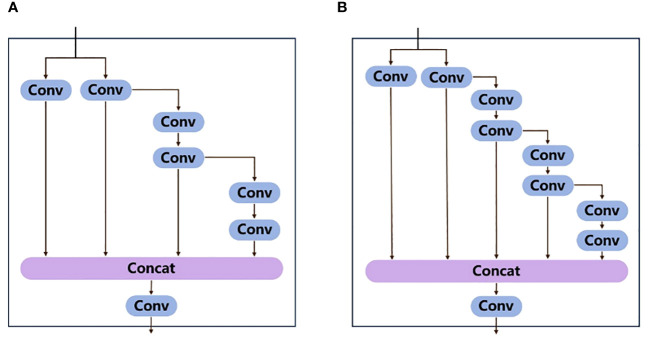
**(A)** ELAN module structure diagram, **(B)** ELAN module structure diagram obtained after scaling and expanding based on ELAN.

The backbone consists of four convolutional layers, four ELAN modules, and three MP modules. Each convolutional layer consists of Convolution, Batch normalization, and SiLU activation function. Features of the input image are extracted, and three feature maps of different sizes are output. The ELAN module enables the deep network to achieve effective learning and convergence by controlling the shortest and longest gradient paths (Wang et al., 2022b). **Figure 5A** shows the structure diagram of ELAN module. A deeper ELAN module in YOLOv7x is obtained by model scaling based on ELAN, as shown in **Figure 5B**.

**Figure 6:**
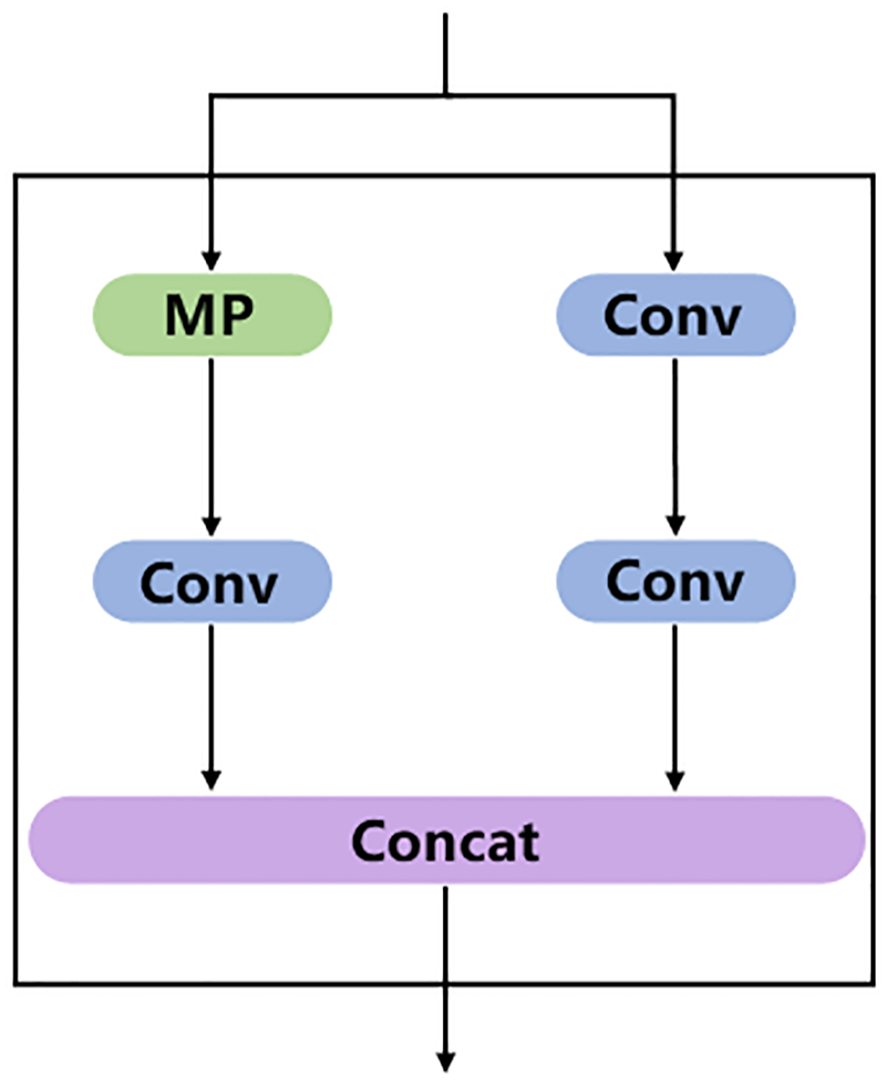
MP module structure diagram, where the MP part is the Max pooling layer.

MP module is divided into two branches and the module function is to carry out downsampling. The structure diagram of MP module is shown in **Figure 6**. The first branch goes through a Max pooling layer for downsampling calculation, and then goes through a Conv layer to change the number of channels. The second branch is downsampled by two Conv layers with different kernel sizes and different synchronization lengths. The final downsampling result is obtained by concatenating the results of the two branches.

The Head part is mainly composed of SPPCSPC module, Conv, Upsampling, MP module, and ELAN module. The Head part extracts features from the three feature maps output by the backbone, and then outputs three feature maps of different sizes. Finally, the anchor is used to predict the location, size, and category of the object in the input image. The prior box is refined by non-maximum suppression to improve the accuracy of model detection.

**Figure 7:**
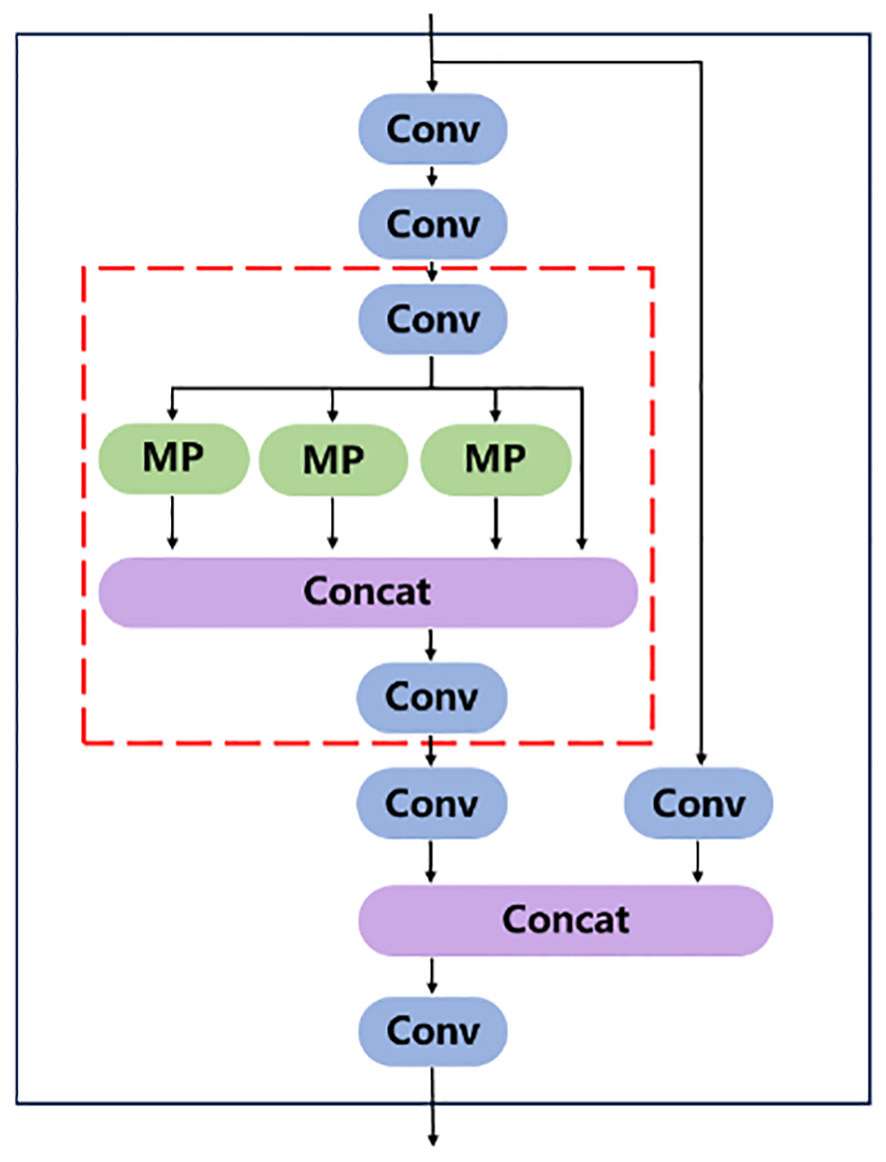
SPPCSPC module structure diagram. The red box in the figure is the SPP structure.

SPPCSPC is used to enhance the expressive power of convolutional neural networks. It is composed of Spatial Pyramid Pooling (SPP) module (He et al., 2015) and Cross-stage Local Network (CSP) module (Wang et al., 2020). SPP uses Max pooling to obtain different receptive fields to adapt to images with different resolutions. **Figure 7** shows the module structure diagram of SPPCSPC, in which the red box part is a block of SPP. In the first branch of the figure, four pooling operations with different kernel sizes are carried out to obtain four different receptive fields to distinguish targets of different sizes. CSP module can improve the representation ability of features and enhance the perception ability of the model to different scales and semantic information. CSP divides the feature map into two parts, one of which is processed conventionally, and the other is processed by SPP. The two parts are merged to improve the speed and accuracy.

#### 2.3.2 SPDConv

**Figure 8:**
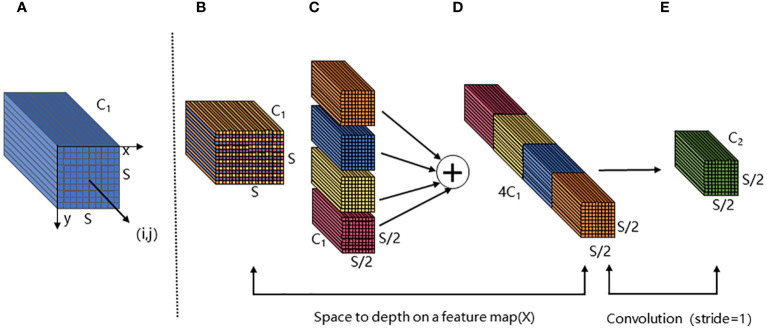
The structure of SPD module. **(A, B)** are the input feature map X. The size of X is S×S, and the number of channels is C_1_. **(C)** is the four submaps of size S/2×S/2 obtained after feature mapping. **(D)** is the output result obtained by splicing the four submaps along the channel. Finally, the output feature map **(E)** is obtained after a 1×1 convolution layer.

SPDConv consists of an SPD layer and a 1×1 convolutional layer. The SPD component generalizes a raw image transformation technique (Sajjadi et al., 2018) to downsample the feature maps within and across the CNN. For the feature map X, when scale is equal to 2, the four sub-maps $f_{0,0}$ , $f_{0,1}$ , $f_{1,0}$ , $f_{1,1}$ obtained by SPD feature mapping. Each sub-map has the shape ( $\frac{S}{2}$ , $\frac{S}{2}$ , $C_{1}$ ), as shown in **Figures 8A–C**. Then, the four submaps are connected along the channel dimension to obtain the feature map X_1_ (**Figure 8D**). The whole process reduces the spatial dimension of X by a scale factor and increases the channel dimension by a scale^2^ factor. A 1×1convolutional layer is added after the SPD layer, so that the output can retain more feature information (**Figure 8E**).

#### 2.3.3 NWD Loss

When the target is small, no overlap occurred between the prediction box P and the Ground-truth box G, or P completely contains G. Intersection over Union (IoU)-Loss cannot provide gradients for network optimization, and vice versa. The problem that the IoU-based loss function sensitive to the position deviation of small targets can be solved and gradients for network optimization can be provided by using Normalized Gaussian Wasserstein Distance (NWD) to design the Loss function NWD Loss (Wang et al., 2021). NWD first models the bounding box as a two-dimensional Gaussian distribution, and then uses Wasserstein distance to measure the similarity between these two distributions instead of IoU. The advantage is that even if no/little overlap between two boxes, the similarity can be measured. Furthermore, NWD is not sensitive to the scale of the target and is more stable for small targets.

The bounding box is modeled by Gaussian. In the horizontal bounding box R = (cx, cy, w, h), (cx, cy), w and h represent the center coordinate, width, and height of the detection box, respectively. The bounding box can be modeled as a 2D Gaussian distribution N (μ, Σ) using Equation 1, where μ and Σ denote the mean vector and the covariance matrix of the Gaussian distribution.


(1) $$\begin{array}{c} \mu =\left[\begin{array}{c} c_{x}\\ c_{y} \end{array}\right],\;\Sigma =\left[\begin{array}{cc} \frac{w^{2}}{4} & 0\\ 0 & \frac{h^{2}}{4} \end{array}\right] \end{array}$$

The distribution distance is calculated by the Wasserstein distance in the optimal transportation theory, and then is normalized by the exponential form to obtain the Wasserstein distance of the normalized Gaussian distribution. For two two-dimensional Gaussian distributions ${\text{μ}}_{1}=\text{N}\left({\text{m}}_{1},{\text{ Σ}}_{1}\right)$ and ${\text{μ}}_{2}=\text{N}\left({\text{m}}_{2},{\text{ Σ}}_{2}\right)$ , the Wasserstein distance between ${\text{μ}}_{1}$ and ${\text{μ}}_{2}$ is calculated by Equation 2.


(2) $$\begin{array}{c} W_{2}^{2}\left({\text{μ}}_{1},\;{\text{μ}}_{2}\right)={\left\|{\text{m}}_{1}-{\text{m}}_{2}\right\|}_{2}^{2}+\text{Tr}\left(\Sigma_{1}+\Sigma_{2}-2{\left(\Sigma_{2}^{1/2}\Sigma_{1}\Sigma_{2}^{1/2}\right)}^{1/2}\right) \end{array}$$

The normalized Wasserstein distance obtained by exponential normalization of ${\text{W}}_{2}^{2}\left({\text{μ}}_{1},{\text{ μ}}_{2}\right)$ is expressed as Equation 3, where C is a constant closely related to the data set.


(3) $$\begin{array}{c} \text{NWD}\left({\text{N}}_{\text{a}},\;{\text{N}}_{\text{b}}\right)=\text{exp}\left(-\frac{\sqrt{{\text{W}}_{2}^{2}\left({\text{N}}_{\text{a}},{\text{N}}_{\text{b}}\right)}}{\text{C}}\right) \end{array}$$

NWD is designed as a loss function as shown in Equation 4, where ${\text{N}}_{\text{p}}$ is the Gaussian distribution model of the predicted box and ${\text{N}}_{\text{g}}$ is the Gaussian distribution model of the true box.


(4) $$\begin{array}{c} {\text{L}}_{\text{NWD}}=1-\text{NWD}\left({\text{N}}_{\text{p}},{\text{N}}_{\text{g}}\right) \end{array}$$

### 2.4 Model training

The required hardware environment for training is Intel(R) Xeon(R) Gold 6246R CPU @3.40GHz, NVIDIA Quadro RTX8000 (48GB video memory), and 128GB running memory. The software environment is Windows 10 operating system, and the deep learning model is constructed based on Pytorch1.10 and cuda11.3. During training, the input image size is 640×640, the batch size is 16. The epochs are 150, and the learning rate is 0.01. The optimizer is SGD, and the weight decay coefficient is 0.05. Adding the SPDConv module to the end of YOLOv7x does not change the network structure, so the pre-trained model YOLOv7X.pt provided by official YOLOv7 can be directly used.

### 2.5 Model construction of real-time wheat ear counting

#### 2.5.1 Position prediction

The construction of wheat real-time counting model includes three steps: position prediction, matching tracking, and counting. The Kalman filter is a linear filter for the state transition prediction problem. The state of the object can be represented by a matrix. Two steps are used with state prediction and state update. The state of the wheat in the current frame was used to predict the state of the wheat in the next frame, called state prediction. The state in the current frame is used to update the state of the wheat in the next frame. The whole process is repeated with the change of the frame number, and called state update.

In state prediction, the state of an object is represented by a matrix x. The state matrix is a two-dimensional column vector represented by position P and velocity V, denoted by ${\text{x}}_{\text{t}}=\left[\begin{array}{c} {\text{P}}_{\text{t}}\\ {\text{V}}_{\text{t}} \end{array}\right]$ , and t is the time. The state of the object at a certain time has a linear relationship with the state at the current time, and is expressed as Equation 5. F represents the state transition matrix. B is the control matrix, and is used to represent the way that the control quantity U acts on the current state. When predicting the state of the object, there are uncertain factors called noise. The covariance matrix (Equation 6) is used to represent the existing noise, where P is the covariance matrix representing the noise. Because the prediction model also has noise, the covariance matrix Q is used to represent the noise in the model. The matrix z is used to represent the observed state of the object. The measured state of the object has a linear relationship with the observed state, expressed as Equation 7, where H represents the relationship between the observed and the measured state, and V is the observation noise.


(5) $$\begin{array}{c} {\hat{\text{x}}}_{\text{t}}^{-}={\text{F}}_{\text{t}}{\hat{\text{x}}}_{\text{t}-1}+{\text{Bu}}_{\text{t}-1} \end{array}$$

(6) $$\begin{array}{c} {\text{P}}_{\text{t}}^{-}=F{\text{P}}_{\text{t}-1}{\text{F}}^{\text{t}}+Q \end{array}$$

(7) $$\begin{array}{c} {\hat{\text{z}}}_{\text{t}}={\text{H}}_{{\text{x}}_{\text{t}}}+V \end{array}$$

The state update is represented in Equation 8, where K is the Kalman coefficient. The calculation method of K is in Equation 9. R is the covariance matrix of the observation noise, which needs to be updated after the state update, expressed in Equation 10.


(8) $$\begin{array}{c} {\hat{\text{x}}}_{\text{t}}={\hat{\text{x}}}_{\text{t}}^{-}+{\text{K}}_{\text{t}}\left({\text{z}}_{\text{t}}-{\text{H}\hat{\text{x}}}_{\text{t}}^{-}\right) \end{array}$$

(9) $$\begin{array}{c} {\text{K}}_{\text{t}}={\text{P}}_{\text{t}}^{-}{\text{H}}^{\text{T}}{\left({\text{HP}}_{\text{t}}^{-}{\text{H}}^{\text{T}}+\text{R}\right)}^{-1} \end{array}$$

(10) $$\begin{array}{c} {\text{P}}_{\text{t}}=\left(\text{I}-{\text{K}}_{\text{t}}\text{H}\right){\text{P}}_{\text{t}}^{-} \end{array}$$

#### 2.5.2 Matching and tracking

**Figure 9:**
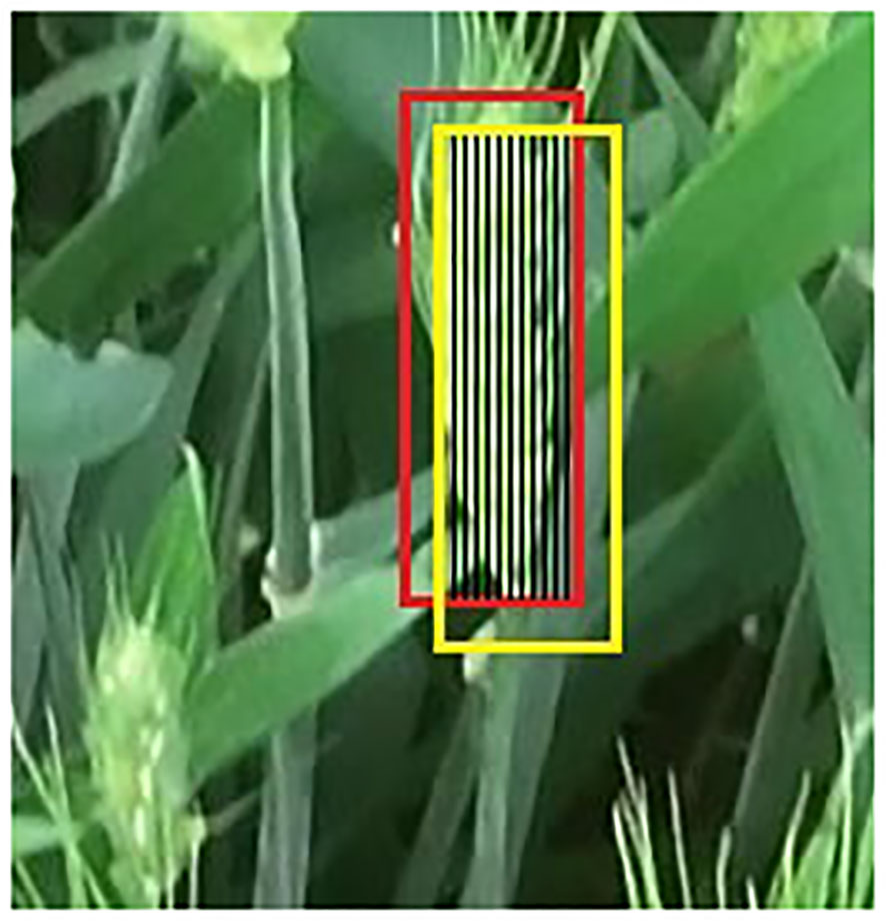
Illustration of the matching rules. The red box is the detection box. The yellow box is the prediction box, and the black shaded part is the IoU of the detection box and the prediction box.

The Hungarian matching algorithm is used to solve the matching problem between the predicted value of the Kalman filter and the detected value in the next frame. The Intersection over Union (IoU) ratio was calculated between the predicted value of the Kalman filter and the detection result of YOLOv7xSPD to determine whether the prediction box and the detection box were the same ear. The IoU threshold was set to 0.7. When the IoU was greater than the threshold, the detection box and the prediction box were classified as the same ear, means that the tracking was successful. **Figure 9** shows the rules whether the wheat ear belongs to the same ear between two frames. The red box is the detection box of YOLOv7xSPD. The yellow box is the prediction box of Kalman filter. The black shadow part is the IoU between the detection box and the prediction box. The tracking is successful when the IoU is greater than 0.7.

#### 2.5.3 YOLOv7xSPD Counter

**Figure 10:**
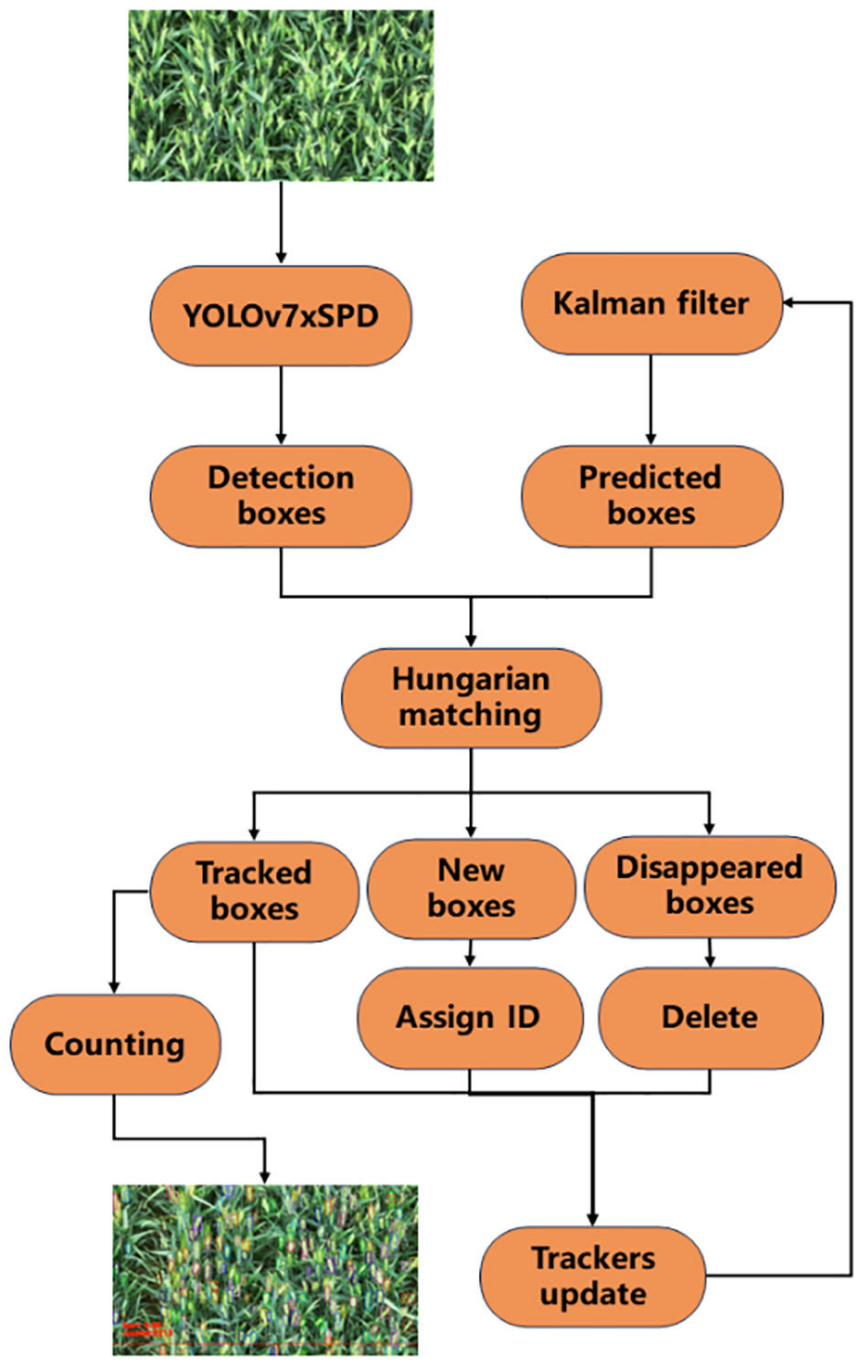
Flowchart of the real-time wheat ear counting. The video image is first input in YOLOv7xSPD to get the detection boxes. Kalman filter is used to follow the tracking boxes and new boxes. The image in the lower left corner shows the effect of counting.

The counting process of YOLOv7xSPD Counter is shown in **Figure 10** with four steps: detection, matching, counting, and updating. Firstly, YOLOv7xSPD is used to detect wheat ear video frame by frame, obtaining the wheat ear detection boxes. The Kalman filter tracking algorithm is then used to predict these detection boxes and obtain the prediction boxes. Secondly, the Hungarian matching algorithm is used to perform IoU matching on the detection boxes and prediction boxes. When the first frame is detected, there are only detection boxes and no prediction boxes, and the matching results only have newly appeared detection boxes. Then, they are assigned IDs and the Kalman filter tracking algorithm is used to predict these wheat ear detection boxes. Starting from the second frame, the Hungarian matching algorithm is used to match the detection boxes and prediction boxes, obtaining successfully tracked wheat ear detection boxes, newly appeared wheat ear detection boxes, and disappeared wheat ear detection boxes. Thirdly, count the successfully tracked wheat ear detection boxes when they pass through the baseline. Meanwhile, assign IDs to the newly appearing wheat ear detection boxes and delete the missing wheat ear detection boxes. Finally, repeat the above steps until the detection is complete.

**Figure 11:**
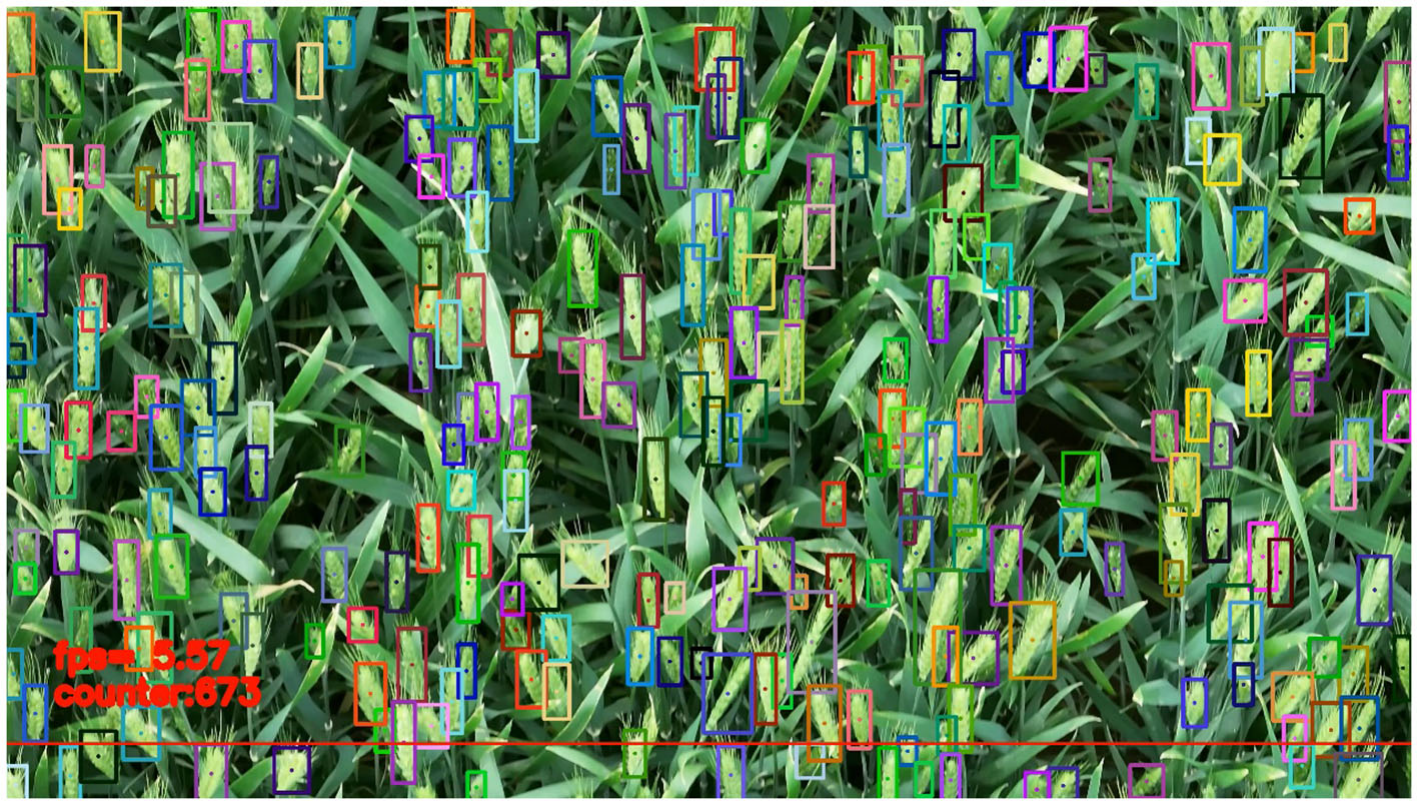
Wheat ear counting with video stream. The detection boxes with different colors represent the detected wheat ears with different IDs. The point in each box is the center point of the detection box. The lower red line is the baseline for counting. When the center point of the detection box passes through the baseline, the number of detection frames and the counting results are shown in the lower left corner.

When the wheat ears appear at the edge of the image, the size of the detection box will change with the appearance of the ears, and the accuracy will be affected with the tracker. Therefore, a baseline is set at the bottom of the image with red color in **Figure 11**. When the wheat ears in the video pass through the baseline, the wheat ears will be counted to avoid the incomplete shape of the wheat ears in the video and the repeated counting.

### 2.6 Model evaluation

Precision (P), Recall (R), F1 Score, and Average Precision (AP) are used to evaluate the model and defined as Equations 11–14:


(11) $$\begin{array}{c} \text{P}=\frac{\text{TP}}{\text{TP}+\text{FP}} \end{array}$$

(12) $$\begin{array}{c} \text{R}=\frac{\text{TP}}{\text{TP}+\text{FN}} \end{array}$$

(13) $$\begin{array}{c} \text{F}1=\frac{2\left(\text{P}\times \text{R}\right)}{\text{P}+\text{R}} \end{array}$$

(14) $$\begin{array}{c} \text{AP}=\int_{0}^{1}\text{P}\left(\text{R}\right)\text{dR} \end{array}$$

True Positives (TP) means that the prediction is positive and correct. True Negatives (TN) means that the prediction is negative and correct. False Positives (FP) means that the prediction was positive and wrong. False Negatives (FN) are that the prediction is negative and wrong. AP is the area of the region enclosed by the curve and the coordinate axis in the PR curve.

The correlation between the model counting and the ground truth number is evaluated by the determination coefficient R^2^ in Equation 15, where $y_{i}$ represents the ground truth number observed manually. ${\hat{\text{y}}}_{\text{i}}$ represents the model counting result. ${\bar{\text{y}}}_{\text{i}}$ represents the average number.


(15) $$\begin{array}{c} {\text{R}}^{2}=1-\frac{\sum_{\text{i}}{\left({\hat{\text{y}}}_{\text{i}}-{\text{y}}_{\text{i}}\right)}^{2}}{\sum_{\text{i}}{\left({\bar{\text{y}}}_{\text{i}}-{\text{y}}_{\text{i}}\right)}^{2}} \end{array}$$

Root mean square error (RMSE) is used to evaluate the model counting and the degree of difference between ground truth number and defined as Equation 16. n is the number of images, ${\text{y}}_{\text{i}}$ is ground truth number, ${\hat{\text{y}}}_{\text{i}}$ represents model counting results.


(16) $$\begin{array}{c} \text{RMSE}=\sqrt{\frac{\sum_{\text{i}}^{\text{n}}{\left({\hat{\text{y}}}_{\text{i}}-{\text{y}}_{\text{i}}\right)}^{2}}{\text{n}}} \end{array}$$

## 3 Result

### 3.1 Wheat ear detection

#### 3.1.1 Model comparison

In all detection models, the IoU threshold and confidence threshold of NMS of each model are set to 0.7 and 0.5 to obtain better detection effects. **Tables 1** and **2** shows the various evaluation results of the six models. The AP and FPS of YOLOv7 and YOLOv7x were similar (94.77% vs. 93.32%, **Table 1**, 6.3FPS vs. 6.7FPS, **Table 2**). However, YOLOv7 consumed 19.5GB more GPU memory compared to YOLOv7x (39.2GB vs. 19.7GB) during model training. Considering hardware costs, YOLOv7x was chosen for improvement to obtain YOLOv7xSPD with higher AP and lower training costs.

**Table 1:** Descriptions of precision, Recall, F1 Score, and AP of Faster RCNN, SSD, YOLOv5s, YOLOv7, YOLOv7x, and YOLOv7xSPD.

Model	P (%)	R (%)	F1 (%)	AP (%)
Faster RCNN	95.54	83.97	89.56	84.58
SSD	92.53	41.69	57.48	55.67
YOLOv5s	95.61	90.49	92.98	92.03
YOLOv7	95.23	**94.90**	95.06	94.77
YOLOv7x	**96.21**	93.47	94.82	93.32
YOLOv7xSPD	95.85	94.71	**95.28**	**94.99**

The bolded values represent the best values for each metric.

**Table 2:** Descriptions of the parameters, FLOPs, FPS, and Training memory occupation of Faster RCNN, SSD, YOLOv5s, YOLOv7, YOLOv7x, and YOLOv7xSPD.

Model	Param	FLOPs	FPS	Training memory occupation (GB)
Faster RCNN	41.4M	12.1G	1.5	23.8
SSD	13.0M	1.5G	5.5	5.8
YOLOv5s	07.1M	16.3G	4.8	6.49
YOLOv7	36.5M	103.2G	6.3	39.2
YOLOv7x	70.8M	188.0G	**6.7**	19.7
YOLOv7xSPD	72.5M	184.8G	6.5	15.0

**Figure 12:**
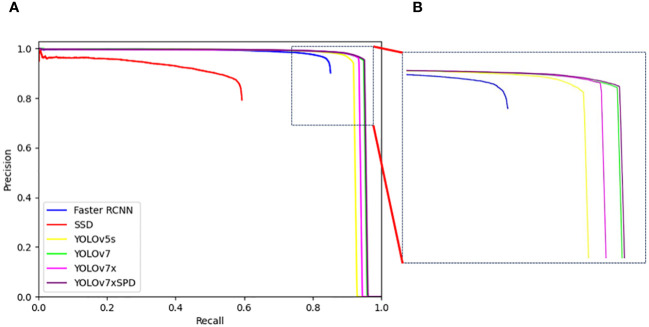
**(A)** PR curves of Faster RCNN, SSD, YOLOv5s, YOLOv7, YOLOv7x and YOLOv7xSPD. **(B)** The local enlarged image represented by box with the dotted line for the difference between the PR curves of YOLOv7 and YOLOv7xSPD.

YOLOv7xSPD performed well in various indicators. Recall, F1 Score and AP were 94.71%, 95.28% and 94.99%, respectively, increased by1.24%, 0.46% and 1.67% compared with YOLOv7x. The various indicators of YOLOv5s were slightly lower than YOLOv7x. Faster RCNN and SSD have a large number of missed detections with low Recall. The detection accuracy of YOLOv7xSPD and YOLOv7 were similar (95.85% vs. 95.23%, **Table 1**), but the two models occupied a significant difference in memory usage during training (15.0GB vs. 39.2GB, **Table 2**). The parameters and FLOPs of YOLOv7xSPD were 72.5M and 184.8G, respectively. Compared to YOLOv7x, its parameters increased by 1.7M and FLOPs decreases by 3.2G. It indicated that the model maintains a reasonable computational scale while achieving superior performance. **Figure 12** shows the PR curve of the six models. The AP of YOLOv7xSPD is the highest, and YOLOv7 is close to YOLOv7xSPD.

**Figure 13:**
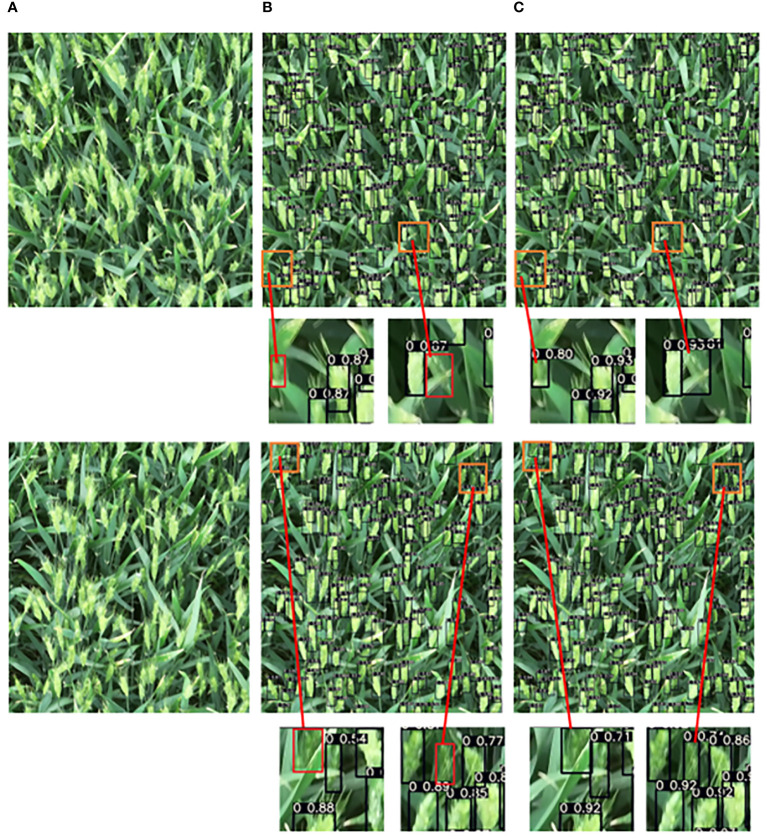
Comparison of the detection result of the wheat ear between YOLOv7xSPD and YOLOv7x. **(A)** original image, **(B)** wheat ear detection with YOLOv7x, **(C)** wheat ear detection with YOLOv7xSPD. Red boxes in B represent the wheat ears not detected by YOLOv7x, which can be accurately detected in C.

Two images were selected from the results to show the detection effect between YOLOv7xSPD and YOLOv7x (**Figure 13**). YOLOv7x has missed detection of small-sized wheat ears, while YOLOv7xSPD can detect small-sized wheat ears compared with that of YOLOv7x, indicating that YOLOv7xSPD can reduce the missed detection rate of small wheat ears and ensure the detection accuracy under large-resolution images.

YOLOv7xSPD achieved the highest F1 Score and AP, indicating that it has the most superior wheat ear detection boxes among all. The recall increased by 1.24% compared to YOLOv7x, indicating that YOLOv7xSPD has a lower miss detection rate. This improvement is reflected in **Figure 13**. In summary, for wheat ear counting tasks, the YOLOv7xSPD model is more suitable.

#### 3.1.2 Cross-validation evaluation

To further verify the robustness of YOLOv7xSPD, 5-fold cross validation was used for training and testing, as shown in **Table 3**. The highest AP is 94.99%, and the lowest is 94.52%. The average of Precision, Recall, F1 Score, and AP are 95.14%, 94.49%, 94.81%, and 94.74%, respectively, and all results are close to the average. The standard deviations (SD) are 1.01%, 0.40%, 0.60%, and 0.20%, respectively, all of which are very small.

**Table 3:** Describes the precision, recall, F1 Score, and average precision of the 5-fold cross validation test results for YOLOv7xSPD, as well as the average and standard deviation (SD) of these indicators.

	P (%)	R (%)	F1(%)	AP (%)
1st fold	95.85	94.71	95.28	**94.99**
2nd fold	94.35	94.06	94.20	94.91
3rd fold	**96.56**	94.71	**95.62**	94.71
4th fold	94.27	94.94	94.60	94.61
5th fold	94.71	94.07	94.39	94.52
Average	95.14	94.49	94.81	**94.74**
SD	1.01	0.40	0.60	**0.20**

The bolded values represent the best values for each metric.

### 3.2 Wheat ear counting

**Figure 14:**
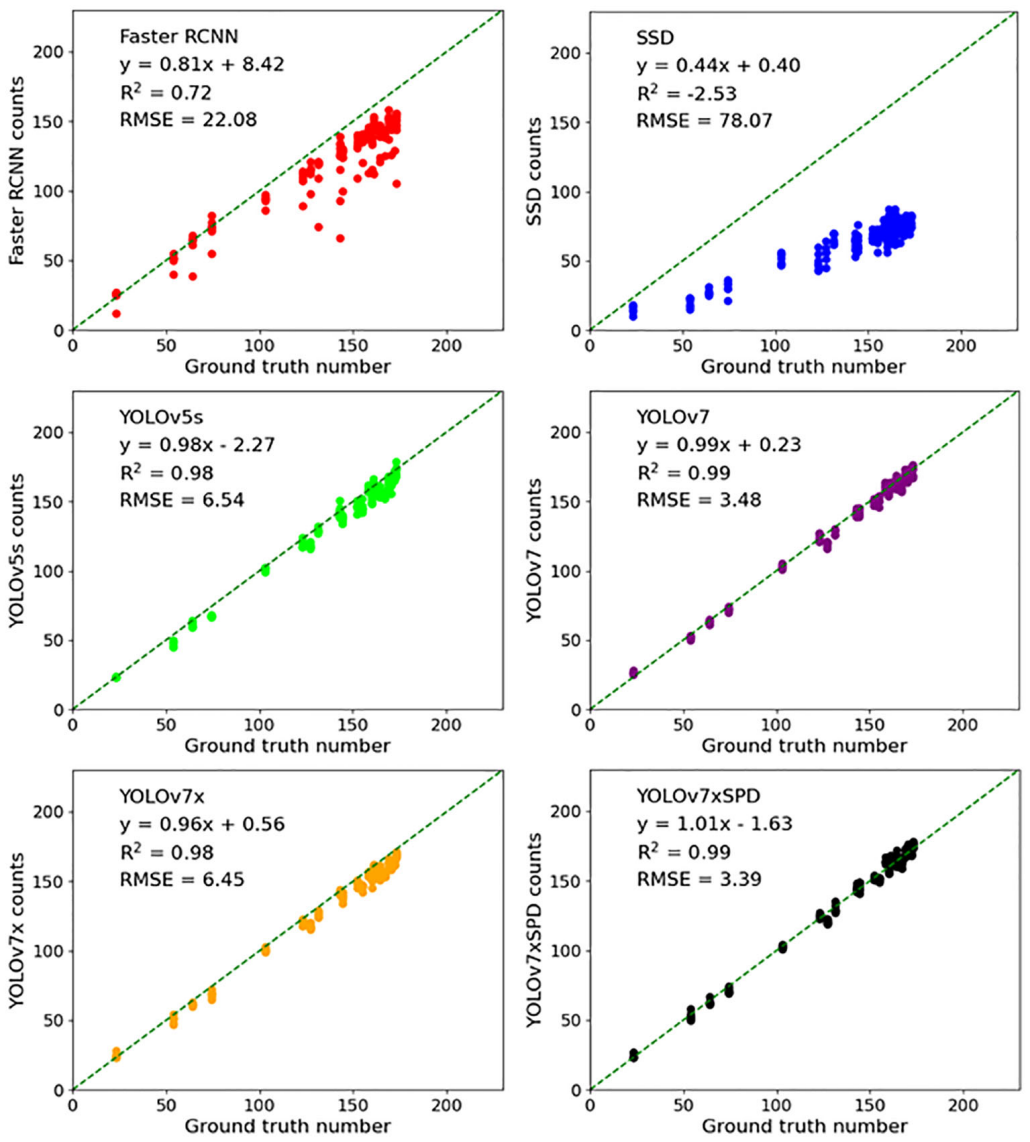
Six detection models were used to perform regression analysis, RMSE and R^2^ calculation on the counting results of the test set.

Six detection models were used to perform regression analysis and RMSE calculation on the counting results of the test set (**Figure 14**). Faster RCNN and SSD have a large number of missed detection. The counting results are R^2^ = 0.72, RMSE=22.08, poorly correlated with the ground truth number as the recall rate of SSD evaluation results is 41.69%, resulting in a large number of missed detection in the model with R^2^=-2.53, RMSE=78.07. The counting results of YOLOv5s performed well, R^2^ only differed from YOLOv7xSPD by 0.01 (R^2^ = 0.98 vs. R^2^ = 0.99), and RMSE differed from YOLOv7xSPD by 3.15 (RMSE=6.54 vs. RMSE=3.39). Average precision and detection speed of YOLOv5s are slightly lower than those of YOLOv7xSPD (92.03% vs. 94.99%, 4.8FPS vs. 6.5FPS, **Table 2**). The counting results of YOLOv7 and YOLOv7xSPD are highly correlated with the ground truth number (R^2^ = 0.99), RMSE were 3.48 and 3.39, respectively, but with high memory occupation during training for the former compared with that for the latter (39.2GB vs. 15.0GB, **Table 2**). YOLOv7x performs well in counting results. According to **Figures 13**, **14**, YOLOv7xSPD has better counting results than YOLOv7x (R^2^ = 0.99 vs. R^2^ = 0.98, RMSE=3.39 vs. RMSE=6.45). Therefore, YOLOv7xSPD can compensate for the missed detection problem for the smaller targets caused by YOLOv7x detection.

**Figure 15:**
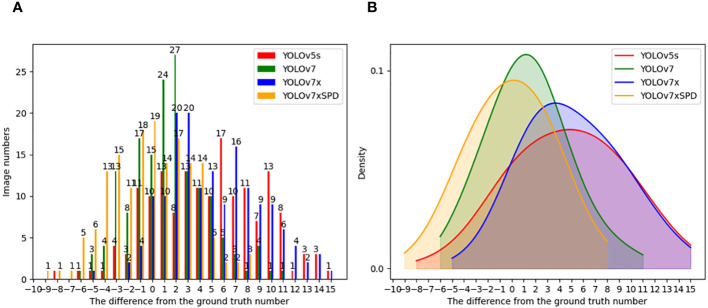
Histogram **(A)** and density **(B)** of the number of gaps between the counting results of YOLOv5s, YOLOv7, YOLOv7x and YOLOv7xSPD and the ground truth number.

**Figure 15** shows histogram (**Figure 15A**) and density (**Figure 15B**) of the number of gaps between the counting results of YOLOv5s, YOLOv7, YOLOv7x and YOLOv7xSPD and the real results. The X-axis represents the gap between the counting results of the model and the ground truth number, and the Y-axis represents the number of images this gap occurred in the test image. Most of the absolute missed detection of YOLOv7 and YOLOv7xSPD is less than 4 with 132, 135 images found respectively. Close to half of the images occurred absolute missed detection within number 0~4 by YOLOv5s and YOLOv7x with 74 and 77 images found respectively. When the absolute missed detection is greater than 8, one and six images were found with YOLOv7xSPD and YOLOv7, but 36 and 34 images for YOLOv5s and YOLOv7. The corresponding density curves were shown in **Figure 15B** YOLOv7xSPD and YOLOv7 are more inclined towards overcounting, while YOLOv5s and YOLOv7x are prone to undercounting. The overcounting of YOLOv7xSPD produced small errors, mainly concentrated between 0 and 4, and the comprehensive counting results are closer to the ground truth number.

**Figure 16:**
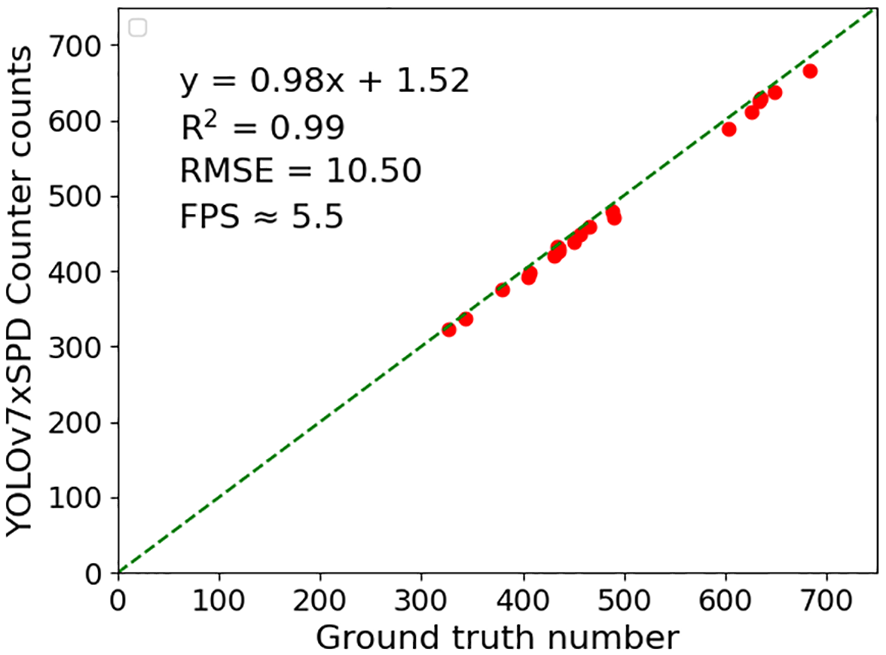
YOLOv7xSPD Counter were used to perform regression analysis, RMSE and R^2^ calculation on the counting results of 20 video test set.

YOLOv7xSPD Counter were used to perform regression analysis and RMSE calculation on the counting results of 20 video test set (**Figure 16**). High correlations are found with R^2^ = 0.99, RMSE=10.05 and the frame rate of counting is about 5.5FPS.

## 4 Discussion

### 4.1 wheat ear detection

Wheat ear number at the early stage of wheat grain filling is closely related to yield and is often regarded as the ideal period for yield estimation (Hernandez et al., 2015; Hassan et al., 2019). The number of pixels of the wheat ear in the large-resolution images collected with UAV are only between 30×80 and 60×100, which increases the difficulty of the wheat ear detection task (Ma et al., 2022). A large number of missed detections occurred by SSD and Faster RCNN model for large wheat ear images with resolution 1920×2160. The detection results of YOLOv5s and YOLOv7x missed detection occurred for the small-sized wheat ear. This is similar to the study of Wu et al. in (Wu et al., 2023). The AP of YOLOv7 is close to YOLOv7xSPD (94.77% vs. 94.99%), and the video memory occupancy of YOLOv7 during training was much higher than that of YOLOv7xSPD (39.2GB vs. 15.0GB). Considering model hardware cost, our newly developed YOLOv7xSPD obtained better wheat ear detection for high-resolution UAV video streams.

The Recall of Faster RCNN and SSD is 83.97% and 41.69%, as Faster RCNN has a weak ability to identify local textures with small-sized object, resulting in missed detection (Cao et al., 2019) The shallow network of SSD has no deep semantic information, thus the detection effect on small targets is poor with lower Recall (Liu et al., 2021). All YOLO series models have AP values more than 90%, as the YOLO series models adjust the size and aspect ratio of the prediction box to accommodate targets of different sizes and shapes (Mahendrakar et al., 2022).

SPDConv module is used to strengthen the feature extraction ability of small wheat ears. NWD Loss function can prevent the loss of small targets by changing the calculation method of loss function. The increase of Recall and AP is consistent with the fact that YOLOv7xSPD can reduce the missing rate of small-sized wheat ears. The CIoU loss function adopted by YOLOv7x needs to calculate the IoU between the two boxes and their center distance. These results were then adjusted for distance and area (Zheng et al., 2021). NWD Loss function adopted by YOLOv7xSPD is to calculate the Wasserstein distance to determine the similarity between the detection boxes and the label boxes. From the perspective of the two loss function theories, the CIoU loss function is more complex, thus YOLOv7x consumes more video memory compared with YOLOv7xSPD during training (19.7GB vs.15.0GB).

### 4.2 wheat ear counting

The ear counting based on the video stream captured by UAV can acquire ear number with a larger unit area, compared to that collected with singe image, and the counting of the wheat ear is not limited by the size of the image (Li et al., 2023). In this study, a real-time wheat ear counting model was constructed based on wheat video streams captured by UAV. A YOLOv7xSPD Counter model was built combined with Kalman filter tracking algorithm (Kalman, 1960) to predict the position of wheat ear in YOLOv7xSPD detection results. The counting effect of using Kalman filter tracking algorithm is consistent with previous research (Yang et al., 2022; Li et al., 2023; Villacrés et al., 2023). The Hungarian matching algorithm (Kuhn, 2010) was used for matching and tracking. In videos with a resolution of 3840×2160, the YOLOv7xSPD Counter detects a frame rate of approximately 5.5FPS. The counting results are highly correlated with manual counting (R^2^ = 0.99), and the RMSE of counting reached 10.05, with nearly real-time counting based on video streams. The counting speed of YOLOv7xSPD Counter is closely related to the resolution of the video and the number of ears, In the calculation experiment, the video resolution is large and the number of wheat ears is between 300 and 700. the higher the resolution and the number of ears, the slower the counting speed.

The detection results of wheat ears directly affect the counting results, as the undetected wheat ears will not be tracked by the Kalman filter tracking algorithm. Due to the influence of turbulence and wind speed, wheat ears sway significantly in UAV videos which causes tracking failure and counting errors (Shi et al., 2021). Therefore, improving the stability of wheat ear tracking by optimizing target tracking algorithms while ensuring the accuracy of the wheat ear detection model will be the main direction of the future research.

In addition, the natural conditions of the field environment (light and wind speed) and the flight status of UAV (speed, altitude, and inclination) can also have an impact on wheat ear detection and counting (Yao et al., 2022). Therefore, increasing training data and optimizing model structure are undertaken to gradually improve the performance and reliability of the model in practical applications.

## 5 Conclusion

The SPDConv module is added to YOLOv7x and the NWD Loss function is used to build a wheat ear detection model YOLOv7xSPD to enhance the detection ability of the model, reduce the occupation of video memory during training. YOLOv7xSPD is then combined with the Kalman filter tracking algorithm to create YOLOv7xSPD Counter to realize real-time wheat counting based on UAV video stream. The conclusions are as follows:

The detection performance of the model (AP=94.99%) is improved with the adoption of SPDConv module and NWD Loss function, and the AP value is 1.67% higher than that of the original YOLOv7x model (94.99% vs. 93.32%). The detection results show that the constructed YOLOv7xSPD model has more advantages to detect smaller wheat ears under large-resolution UAV images. The Kalman filter tracking algorithm is used to track the detection results of YOLOv7xSPD, and the Hungarian matching algorithm is combined to build the YOLOv7xSPD Counter to count the successfully tracked wheat ears. The counting results of 20 videos with YOLOv7xSPD Counter were highly correlated with the ground truth number results (R^2^ = 0.99). The results can provide data support for wheat yield prediction, genetic breeding and optimized planting management research.

## Data availability statement

The original contributions presented in the study are included in the article/supplementary material. Further inquiries can be directed to the corresponding authors.

## Author contributions

XX: Methodology, Writing – original draft, Writing – review & editing, Formal analysis, Project administration, Funding acquisition. LZ: Methodology, Software, Validation, Visualization, Writing – original draft, Data curation, Formal analysis, Project administration, Writing – review & editing. HY: Formal analysis, Methodology, Project administration, Writing – original draft, Conceptualization, Data curation, Writing – review & editing. GS: Data curation, Resources, Writing – original draft, Investigation. SF: Data curation, Resources, Writing – original draft, Investigation. JZ: Writing – original draft, Methodology, Project administration, Writing – review & editing. YM: Writing – original draft, Methodology, Project administration, Writing – review & editing.
